# Antiepileptogenesis and disease modification: Clinical and regulatory issues

**DOI:** 10.1002/epi4.12526

**Published:** 2021-07-29

**Authors:** Jacqueline A. French, Martina Bebin, Marc A. Dichter, Jerome Engel, Adam L. Hartman, Sergiusz Jóźwiak, Pavel Klein, James McNamara, Roy Twyman, Paul Vespa

**Affiliations:** ^1^ NYU Grossman School of Medicine and NYU Langone Health New York NY USA; ^2^ UAB School of Medicine and UAB Epilepsy Center Birmingham AL USA; ^3^ University of Pennsylvania Philadelphia PA USA; ^4^ David Geffen School of Medicine at UCLA and the Brain Research Institute Los Angeles CA USA; ^5^ Division of Clinical Research National Institute of Neurological Disorders and Stroke/NIH Bethesda MD USA; ^6^ Department of Child Neurology Medical University of Warsaw Warsaw Poland; ^7^ Mid‐Atlantic Epilepsy and Sleep Center Bethesda MD USA; ^8^ Department of Neurobiology Duke University School of Medicine Durham NC USA; ^9^ Amron Neuroscience, LLC Darby, MT USA; ^10^ Departments of Neurology and Neurosurgery David Geffen School of Medicine UCLA Los Angeles CA USA

**Keywords:** antiepileptogenesis, clinical trials, disease modification

## Abstract

This is a summary report of clinical and regulatory issues discussed at the 2018 NINDS workshop, entitled “Accelerating Therapies for Antiepileptogenesis and Disease Modification.” The intent of the workshop was to optimize and accelerate development of therapies for antiepileptogenesis (AEG) and disease modification in the epilepsies. The working group discussed nomenclature for antiepileptogenic therapies, subdividing them into “antiepileptogenic therapies” and “disease modifying therapies,” both of which are urgently needed. We use the example of traumatic brain injury to explain issues and complexities in designing a trial for disease‐preventing antiepileptogenic therapies, including identifying timing of intervention, selecting the appropriate dose, and the need for biomarkers. We discuss the recent trials of vigabatrin to prevent onset and modify epilepsy outcome in children with tuberous sclerosis (Epistop and PreVeNT). We describe a potential approach to a disease modification trial in adults, using patients with temporal lobe epilepsy. Finally, we discuss regulatory hurdles for antiepileptogenesis and disease‐modifying trials.

**Key Points**

- There is an urgent clinical need for new treatments that prevent epilepsy development or modify epilepsy after it presents (including preventing increases in seizure frequency, severity, or drug resistance, or actually reducing or eliminating seizures over time, on or off medication).To date, such treatments do not exist.
- While developing novel treatments is critical, it is equally important to develop new paradigms for clinical trials that will be able to identify antiepileptogenic and disease‐modifying effects.
- Prevention of epilepsy after traumatic brain injury is one area of active and potentially fruitful investigation by a number of groups, who aim to develop biomarkers and determine feasibility of trials in specific subsets of patients. Prevention of epilepsy and related comorbidities in children with tuberous sclerosis complex by treating prior to seizure development is also under study.
- As antiepileptogenic and disease‐modifying treatments are developed, a new framework for regulatory labeling will have to be developed in tandem.

## 1 INTRODUCTION

To date, therapies available to treat people with epilepsy are predominantly symptomatic in that they aim to control seizures, rather than specifically address an underlying disease process. While many people can achieve complete control of seizures with these therapies, most require continued treatment for years to decades because these therapies do not address the mechanisms underlying development, progression, or persistence of epilepsy. The socioeconomic impact of chronic epilepsy is substantial, accounting for 0.5% of the global disease burden.^1^ The annual total cost of epilepsy in the United States jumped nearly 200% from $12.5 billion in 1995 to $36.8 billion in 2014.^2^, ^3^ Epilepsy can affect individuals across the age spectrum but because new‐onset epilepsy occurs frequently in the young, especially in individuals with neurodevelopmental disorders, the lifetime healthcare economic impact is substantial. A recent study of new cases of epilepsy in Australia estimated a lifetime cost associated with epilepsy to be over $1.5 million per individual.^4^ A recent analysis of the economic impact of a preventative or disease‐modifying therapy has not been done for epilepsy, but analogous studies in neurodegenerative disease conditions such as Parkinson's disease suggested that even modest impacts on disease incidence or course would have relevant effects on healthcare cost burden.^5^ For the individual, reducing or preventing the cognitive, behavioral, or social impact of chronic epilepsy is also an imperative. Thus, there is an urgent need for new therapies that prevent epilepsy, ameliorate the disease course, or cure the disorder. The National Institute of Neurological Disorders and Stroke (NINDS) recently convened a workshop, entitled “Accelerating Therapies for Antiepileptogenesis and Disease Modification”. The intent of the workshop was to optimize and accelerate development of therapies for antiepileptogenesis (AEG) and disease modification in the epilepsies. This manuscript addresses the clinical aspects of antiepileptogenesis and disease modification research. Two other papers developed through the workshop addressed preclinical issues and biomarkers^6^, ^7^

A recent paper suggested that the term “antiepileptogenesis” should be used both for treatment prior to the development of epilepsy and to interruption of the ongoing process of epileptogenesis after epilepsy manifests.^8^ This definition is scientifically appropriate but problematic when considering therapeutic intervention, as it may be very difficult to disentangle ongoing epileptogenesis from ongoing epilepsy. We will therefore use the term “antiepileptogenesis” as the prevention of epilepsy in someone with a clear, known risk. Risk for epilepsy development syndrome (or RED syndrome) has been proposed as a name for people known to be at risk for epilepsy.^9^, ^10^ The RED score is just a simple and easy way to understand relative risks for lay people, physicians, and non‐statisticians. Almost all risks will be ranges rather than single values—such as 8%–12% or 5%–25% depending on several other factors. A similar terminology was widely adapted for people at risk for developing Parkinson's disease (Parkinson's associated risk syndrome),^11^ or PARS.

Epilepsy disease modification is a therapy that will prevent or retard progression in the seriousness of epilepsy (particularly relevant for known progressive epilepsy syndromes), or lessen or eliminate the manifestations of epilepsy, once it has been established. To what extent this may include some aspect of epileptogenesis, as noted above, remains under study. This includes impact on the ongoing epileptogenic process, if present. Epilepsy disease modification may include preventing increases in seizure frequency, severity, or drug resistance, or actually reducing or even eliminating seizures over time, on or off medication. Of note, not all epilepsies are understood to be progressive diseases, and in these cases, disease modification may actually represent a permanent improvement in the disease or its comorbidities. There are a number of well‐established comorbidities associated with various forms of epilepsy that may be as disabling as the recurrent seizures themselves and these need to be considered in the context of an antiepileptogenic and disease‐modifying therapy. These include cognitive, behavioral, psychiatric, endocrinological, and social issues. A disease‐modifying intervention could also prevent any of these comorbidities, although it would not be called *epilepsy* disease modification unless it also impacted seizures. Restated, disease modification may take many forms with multiple distinct outcomes depending on the nature of the epileptic condition.

Understandably, AEG and disease modification trials will be more complex than symptomatic interventional trials. While many “candidate” interventions have been identified in preclinical models of epilepsy, clinical trial designs (type of epilepsy, time of intervention, specific readouts) to demonstrate disease modification or prevention need to be considered. It is also not clear if any therapy that impacts a single type of epilepsy would be useful in other epileptogenic scenarios. There may be “groups” of risk factors for which a given therapy may prove to be useful, as for example, epileptogenesis after TBI, but whether the mechanisms are generalizable to other etiologies (eg, ischemic or hemorrhagic strokes, status epilepticus, etc) is uncertain. However, further preclinical studies could provide insights into common or generalizable mechanisms. Biomarkers will play an important role in both identifying individuals at risk and as indicators and measures of disease progression and therefore should be an important component of clinical trials. A biomarker is defined as a characteristic that is measured as an indicator of normal biologic processes, pathogenic processes, or responses to an exposure or intervention, including therapeutic interventions. A resource was created by the FDA‐NIH Joint Leadership Council in 2015 called BEST (Biomarkers, EndpointS, and other Tools) that was intended to facilitate use of biomarkers in biomedical research, clinical practice, and medical product development. A recent publication reviewed possible biomarkers, including those to be used in antiepileptogenesis trials.^12^ In addition, a publication emerging from this NINDS antiepileptogenesis conference laid out a strategic roadmap for biomarker development.^7^

In the following sections, we discuss two examples of paths to a clinical trial, one for antiepileptogenesis in adults, another for disease modification in children, to exemplify important issues that should be considered in order to mount a clinical trial. We also provide an option of a clinical trial for a potential disease‐modifying agent in adults. Finally, we make suggestions for the future.

## 2 ANTIEPILEPTOGENESIS

In adults, structural injury to the brain from trauma, hemorrhage, infection, ischemic stroke, surgery, inflammatory/immune diseases are the most frequent insults that initiate epileptogenesis. These injuries are common and account for over two million patients/year being exposed to the risk of epilepsy. Among these structural injuries, certain characteristics of the injury increase the risk of epilepsy, namely brain hemorrhage, severe injury, age, surgical intervention, and others. Acute seizures are common after many of these structural insults.

Studies in humans to prevent epileptogenesis using standard antiseizure drugs after stroke, severe traumatic brain injury, or craniotomy were unsuccessful.^10^, ^13^, ^14^, ^15^, ^16^ The failure of these studies may at least partly be explained by inclusion of patients at different risk of epilepsy, highlighting the need for reliable and clinically applicable biomarkers of epileptogenesis. As will be discussed later in this paper, recently published results of the EPISTOP trial examined disease modification in children with TSC.^17^

### 2.1 Traumatic brain injury

There have been numerous trials of therapies intended to prevent epilepsy after traumatic brain injury (TBI), and as noted above, none has been successful to date.^18^ There are several reasons why TBI, specifically moderate‐to‐severe TBI, is potentially attractive as a disease prevention antiepileptogenesis target.

Traumatic brain injury (TBI) accounts for approximately 6% of all epilepsies.^19^ Post‐traumatic epilepsy (PTE) develops after a latent period of weeks to years.^20^, ^21^, ^22^ Most patients will develop PTE within 2 years of TBI, but mild TBI results in a longer latent period lasting one to two decades in some reports.^20^, ^21^, ^22^, ^23^ The incidence of PTE ranges from 5% to nearly 50% with increasing incidence correlating with more severe injury phenotype, such as brain contusion, skull fracture, or penetrating injury.^24^, ^25^ Most of these will occur within 2 years of injury.

A long latency to seizures and/or a low incidence of epilepsy would lead to substantial feasibility issues for a clinical trial. Thus, moderate‐to‐severe TBI presents the most feasible target. Another rationale for selecting severe TBI patients as subjects for an AEG trial is that the onset of injury is typically well documented. Severe TBI is typically a one‐time event accompanied by medical attention in an inpatient setting, where subjects could be easily identified.

Determining a specific phenotype of moderate‐to‐severe TBI with the highest risk of PTE would therefore be the most logical next step in defining a target patient population for an AEG trial. Specific phenotypic features of moderate‐to‐severe TBI that raise the risk of PTE include hemorrhagic contusion load, depressed skull fractures, low Glasgow Coma Score, craniotomy to remove hematomas, gender, early post‐traumatic seizures, and duration of coma.^23^ A few studies have reported higher risk with certain genetic variants, such as adenosine receptor subtypes.^26^ The severe TBI phenotype including temporal lobe brain hemorrhage and early seizures had the highest incidence of PTE in recent reports.^23^

### 2.2 Biomarkers

Even with moderate‐to‐severe TBI, clinical trials are likely to be too expensive to consider without predictive biomarkers, because the number of subjects necessary to obtain statistical significance is too large and the follow‐up period needed to determine outcome might be too long.

Because patients with moderate‐to‐severe TBI are in an inpatient setting, there is an opportunity to conduct brain imaging, blood sampling, and electroencephalography, each of which may yield important biomarkers. Identification of biomarkers that predict development of epilepsy will be a critically important requirement for future trials of any antiepileptogenic therapy for prevention of epilepsy after TBI. A separate paper from the Antiepileptogenesis Workshop addresses biomarkers.^7^ Planning an economically feasible clinical epilepsy prevention AEG trial could require reliable biomarkers to identify subjects with a high likelihood of developing PTE to enrich the subject population. Equally important would be epilepsy biomarkers to determine whether the treatment is effective without the need to wait for seizures to occur.

This rationale was used to justify the funding of two brain injury biomarker studies. The Epilepsy Bioinformatics Study for Antiepileptogenic Therapy (EpiBioS4Rx) and the TAPTE‐(Team Approach to prevention and treatment of Post‐traumatic Epilepsy) CURE grant seek to establish reliable biomarkers for PTE in animals and humans, and are anticipated to inform our working knowledge of the appearance, evaluation, and progression of biomarkers of PTE.^27^ Large biomarker studies of mostly mild TBI are also nearing completion, and information from TRACK‐TBI^28^ (an NINDS‐funded, multicenter study “Transforming Research and Clinical Knowledge in Traumatic Brain Injury”) and Center‐TBI^29^ (a large European project that aims to improve the care for patients with Traumatic Brain Injury) will likely inform the incidence of PTE within 2 years of milder forms of TBI. Collectively, these studies have raised awareness of PTE among the general public and advocacy groups for TBI recovery. The EpiBioS4Rx public engagement core is specifically addressing methods to enhance patient recruitment and retention for future AEG trials through a prospective plan to engage patients, families, and advocacy groups. The severe TBI population would be a suitable population for early biomarker monitoring, patient selection, and structured clinical trial intervention starting within days to weeks of the TBI. Establishing the appropriate phenotype of brain injury will be necessary prior to embarking on an AEG trial. We propose that a severe injury that includes intracerebral hemorrhage is the ideal phenotype for an AEG trial given the high incidence of PTE occurring after a relatively shorter latent period.

An important gap in knowledge is the current uncertainty of specific biomarkers for epileptogenesis and for epilepsy. However, prospective animal models have begun to identify biomarkers of specific pathways that are involved in epileptogenesis.^27^ The biomarkers need to properly reflect the epileptogenesis process rather than the injury or recovery process. A large number of injury biomarkers have been proposed, including specific cellular subtypes and most of these occur during epileptogenesis. Thus, prospective study of the evolution of these biomarkers to differentiate patterns of biomarkers over time is needed to create reliable indicators of epileptogenesis and epilepsy biomarkers.

### 2.3 Timing and duration of treatment

After establishing reliable biomarkers for epileptogenesis and epilepsy, the time window for treatment and the treatment duration will need to be established. Theoretically, there may be different time windows after injury requiring staging of the process for different types of treatment. Use of a biomarker, such as EEG,^30^ to mark the onset of epileptogenesis may be one method to establish the time window. Establishing the time window based on animal studies is always complicated by interspecies differences in which timing of events occurs more rapidly in animals than in humans. The optimal duration of treatment needs to be determined and, in contrast to clinical trials of antiseizure medications, the duration must be long enough to substantially interrupt the epileptogenic process and for the objective outcome event to be seen in an adequate proportion of patients. Traditional requirements by the US FDA are for objective clinical endpoints such as incidence of seizures. Once validated biomarkers become available, the objective outcome event may be a reduction in a sensitive epilepsy biomarker rather than the occurrence of PTE. Thus, careful consideration will need to be given to a balanced approach between biomarkers that predict outcome and clinical endpoints. The duration will also be informed by the potential adverse effects of the AEG drug on cognition in patients who are recovering from TBI. Hence, a comprehensive evaluation of both on‐target and off‐target effects and side effects of the AEG drug is needed.

## 3 DISEASE MODIFICATION

Currently available pharmacological treatments of epilepsy are only seizure suppressing, and none is disease‐modifying or antiepileptogenic, whether introduced before or after clinical seizures. A number of potential pathogenic targets have been identified in preclinical models^31^ and there have been recent strides to introduce disease‐modifying interventions including small molecules, biologics, gene therapies, and neuronal implants.^32^ It is possible that even antiseizure medicines have disease‐modifying properties, but trial designs currently used for regulatory approval for antiseizure medicines (randomized placebo‐controlled add‐on trials, followed by open‐label extension studies) would not be able to demonstrate disease modification. New trial designs will be needed to demonstrate disease modification.

### 3.1 Disease modification in children

The group of pediatric epilepsies may represent the most appropriate category for disease modification/antiepileptogenic trials. Many pediatric epilepsies have a genetic etiology. Because of their genetic basis, and an early and identifiable epilepsy onset, there is an opportunity for the development of clinical therapeutic treatment strategies that focus on preventative/disease‐modifying therapies. This is a critical starting point for therapeutic interventions given the effects of epileptogenesis on development and cognitive outcomes.

Early diagnosis of the underlying disease may allow therapeutic intervention before clinical seizures, ideally reducing the risk of epilepsy and epileptic comorbidities. Such conditions as tuberous sclerosis complex (TSC) or Sturge‐Weber syndrome (SWS) may be described as the risk for epilepsy development syndromes (or RED syndromes)—conditions with well‐known natural course of disease characterized by frequent and devastating seizures associated with low quality of life of the patients.^33^ In such conditions, prevention or disease modification trials seem to be of utmost importance.

### 3.2 TSC as a genetic model of a preventable or modifiable epilepsy in humans

TSC is an autosomal dominant neurocutaneous disorder that affects approximately 1 in 6000 individuals and is a common genetic cause of catastrophic early childhood epilepsy as well as later onset epilepsy. Pathogenesis of the condition is caused by loss‐of‐function germline mutations in either of the tumor suppressor genes *TSC1* or *TSC2*. These mutations lead to hyperactivation of the mTOR pathway, which was shown to play an important role in epileptogenesis in TSC and other animal models of epilepsy syndromes, such as traumatic brain injury, neonatal hypoxia, or kainate‐induced status epilepticus.^33^, ^34^ A recent double‐blind, randomized, placebo‐controlled trial (EXIST‐3) showed that mTOR inhibitor, everolimus, was significantly more effective than placebo in reducing focal seizures in TSC patients with drug‐resistant epilepsy.^35^

Epilepsy is present in a high percentage of TSC patients, possibly reaching about 90% during their lifespans and in a prospective study of infants followed since neonatal period about 71% of children developed epilepsy in the first 2 years of life.^36^ Epilepsy onset in the neonatal period is reported in about 5%‐6% of children usually with significant cortical malformations meeting the criteria for focal cortical dysplasia (FCD).^37^ Approximately 65% of those with epilepsy have medically refractory epilepsy. In the group of patients with presentation of epilepsy in the first year of life up to 82% develop intellectual disability and autistic behaviors.^37^, ^38^

In recent years, there is an increasing group of fetuses and newborns with a diagnosis of TSC before seizures, creating an opportunity for preventive or disease‐modifying treatment. Prenatal diagnosis is possible due to the diagnosis of cardiac tumors, which are the first visible sign of the disease. Prenatal brain MRI may confirm the diagnosis.^39^

Video‐EEG studies carried out in Europe^36^, ^39^ and in the United States^40^, ^41^ confirmed that regular electroencephalographic recordings may disclose epileptiform activity before seizures and could be used as biomarker for identification of individuals at high risk of epilepsy development. In this study, interictal epileptiform discharges identified impending epilepsy in the majority (77%) of seizure‐naïve infants with TSC.^40^

In a large group of infants followed at The Children's Memorial Health Institute in Warsaw, 70% developed spike and wave complexes or polyspike activity on EEG, which evolved to more generalized activity. Initially, brief focal seizures could be seen in children with multifocal spikes or spike and wave complexes, most frequently in the 4th or 5th month of life.^41^ If not successfully treated, they evolve into infantile spasms.

Early treatment with vigabatrin prior to epilepsy development has been applied in three studies in infants with TSC: the open‐label study published by Jozwiak et al (2011, 2019)^36^, ^42^ as well as two randomized trials: EPISTOP and PreVENT studies. Results of the EPISTOP study have been recently published documenting lower risk of clinical seizures (odds ratio [OR] = 0.21, *P* = .032), drug‐resistant epilepsy (OR = 0.23, *P* = .022), and infantile spasms (OR = 0, *P* < .001) in those treated with vigabatrin prior to overt seizure occurrence.^17^ There are an increasing number of papers demonstrating the utility of pre‐seizure EEG surveillance in TSC as an initial step to preventive or disease‐modifying treatment.^43^, ^44^, ^45^ Similar trials are being considered in Sturge‐Weber syndrome.^46^ Due to the role of epilepsy in the development of neurocognitive disabilities and autism, the lessons learned from TSC are likely to be applicable in other epileptic encephalopathies.^47^

As a result of these trials, early presymptomatic assessment of impending epilepsy with EEG surveillance in newborns and infants with TSC is currently underway in areas of Europe,^48^ and in these centers, sequential video EEG recording has been recommended in every newborn and infant newly diagnosed with TSC, so that treatment with vigabatrin can be initiated immediately after observing electrographic abnormalities or clinical seizures.^49^ Vigabatrin has already been determined to be the first‐choice treatment for focal seizures and infantile spasms in TSC.^50^

Prevention and/or modification of epileptogenesis by inhibiting the mTOR pathway in newborns and infants with TSC are very tempting in light of the vigabatrin results. A new double‐blind, randomized, prospective study VIRAP (EudraCT number 2020‐003231‐19) is going to compare rapamycin vs vigabatrin for prevention of epilepsy in infants with TSC.

### 3.3 Disease modification in adults

A target of many disease‐modifying therapies is the temporal lobe. This is because it is considered to be a common cause of treatment‐resistant epilepsy and also because many animal models of temporal lobe epilepsy exist.^51^ Here guided by insights gained from preclinical studies, and to illustrate these concepts, we briefly describe a disease modification clinical trial approach to guide dosing and toxicology considerations for a clinical candidate and design of a Phase 2a trial. This is one of many likely potential trial designs that could be considered. The purpose of providing it here is to demonstrate that disease modification trials will look very different from antiseizure trials, and novel trial designs will be necessary. The goal would be to determine whether fleeting exposure of patients with medically refractory mesial temporal lobe epilepsy (MTLE) to test drug enhances responsiveness to an anticonvulsant regimen. If such a trial were undertaken, subjects could be identified from patients with medically refractory MTLE who have undergone presurgical evaluation for temporal lobectomy, but before resection. Criteria for patient inclusion that could be considered include the following: (a) focal seizures with or without evolution to bilateral tonic‐clonic with frequency of at least 4/month; (b) MRI evidence of unilateral hippocampal sclerosis; and (c) interictal spikes ipsilateral to the sclerotic hippocampus. Patients would remain on baseline antiseizure medication. Following a baseline period sufficient to ascertain seizure frequency information to allow a post‐treatment control, patients could be treated with either drug or vehicle and treated for the duration of time considered necessary to have an impact on epileptogenesis based on preclinical data. (Treatment with investigational agent should not be continued during the outcome assessment, particularly if the intervention also had the potential to have antiseizure properties). Optimally, drug dose and schedule would be based upon evidence of target engagement using an appropriate biomarker. Seizure frequency would be assessed during baseline, during treatment, and for an outcome assessment period following cessation of treatment (duration for durability of a disease modification effect will need further clarification). Neuropsychological evaluation of cognitive and emotional indices including memory testing could be conducted during baseline, treatment, and post‐treatment. The primary outcome measure might be number of seizures during treatment and the 4 months following treatment vs the baseline period. A biomarker might be built in as a substudy to possibly provide supportive and potentially independent evidence of a therapeutic effect on the underlying disease process. Secondary outcome measures might include neurocognitive and behavioral measures. The criterion for success could be that, in comparison with placebo, one or more patients with TLE became medically responsive following treatment or that seizure frequency was reduced. A paired design with each patient serving as his or her own control could be used.

## 4 POTENTIAL MAJOR ADVANCES ON THE HORIZON

A number of recent advances in the field may increase the likelihood of a successful development of an antiepileptogenic or disease‐modifying therapy in the future. For one, we have the tools to perform better natural history studies. This will be important both for planning antiepileptogenesis trials, where using large data sets will permit definition of subpopulations at risk for developing epilepsy and for stratifying risks, but also for disease‐modifying therapies, where it will be vital to understand the slope of worsening, if any, and the likelihood of spontaneous remission. There are also very active biomarker discovery programs using a variety of modalities (imaging, biochemical, molecular) to identify biomarkers of the process of epileptogenesis that ultimately may be employed, first for validation, and then for assessment of clinical trials. In addition, the technology and human experience of chronic intracranial recordings have progressed rapidly and may soon be available as an acceptable means of monitoring therapeutic interventions designed to prevent or ameliorate epilepsy.

## 5 REGULATORY CONSIDERATIONS AND LABELING

It is important for labeling of an approved pharmaceutical product to appropriately and adequately inform patient and prescriber regarding the intended and proper use of the drug product and its known safety profile. Labeling also provides the basis for claims around product differentiation and commercial promotion (ie, how the product performs differently from other therapeutic agents and what language limitations exist for promoting or selling the product). For therapeutic interventions that target the underlying disease, observed data must demonstrate and prove effects on the disease. For example, does the agent modify the course of disease progression (ie, slow the course of disease) or “prevent” the disease (ie, delay onset of disease or decrease risk of disease development)?

The area of oncology has established labeling language that provides rather straightforward interpretation of the effects of interventions that modify the disease. For example, “5‐year survival rate” or “percent disease free at 5 years” are easily interpreted by both patient and prescriber, and these claims can be supported by well‐established regulatory approved clinical trial designs and outcome measures. Indication claims may be rather general, such as “indicated for the treatment of xxx cancer,” but may also claim prevention and reduced risk of cancer. For example, the National Cancer Institute considers raloxifene to be one of two compounds that can prevent breast cancer and Evista (raloxifene) has indication claims for treatment and prevention of osteoporosis and reduction of risk in invasive breast cancer.^52^ Data from several clinical trials are outlined in the Evista FDA‐approved label and provide the evidence supporting these claims and label language.

The area of neuroscience is beginning to establish labeling claims and paths for marketing authorization claims for disease‐modifying or prevention therapies. Labels exist already that provide claims for disease modification in multiple sclerosis (MS) and the FDA draft guidance for early Alzheimer's disease provides a regulatory framework for identifying/diagnosing early disease individuals and consideration of clinical trials designs and endpoints.

In MS, the term disease‐modifying therapy (DMT) is specifically called out in promotional advertisements and references to therapeutic effects using language such as “demonstrated to delay disability progression” and “xx% of patients remained free from disability progression” can be found. This information is provided with indication claims that only refer to the treatment of relapsing forms of MS, but can also specify the MS subtypes such as secondary progressive. As in oncological drug development, clinical trial data substantiate these claims and disease modification language, but notably most MS trials use a community recognized and commonly accepted disability progression scale.^53^

Epilepsy encompasses a multitude of conditions with diverse causes and different clinical courses. Overall, the heterogeneity of conditions does not differ much from cancer or neurodegenerative disease, but development of antiepileptogenic and disease‐modifying therapies will require a solid understanding of associated causal and risk factors and parameters, including biomarkers, that change over time in the natural history of disease development and disease course.

The regulatory precedents described above suggest that labeled indications for epilepsy DMTs can potentially be generalized and language describing epilepsy disease modification or prevention contained within the label could be more specific. Like other fields, disease modification claims for epilepsy therapies will require substantial evidence for these claims established by appropriate and regulatory acceptable clinical trial designs with relevant outcomes measures. Opportunities exist for developing FDA guidelines, perhaps with novel endpoints encompassing the scope of progressive disease burden such as specific comorbidities or extending to disease modification therapies with easily interpretable outcomes such as “x year seizure‐free.” Epilepsy community experts will need to drive consensus opinions and white papers on recommended clinical study designs and outcome variables as in this paper from the NINDS Antiepileptogenesis 2018 Workshop.

## 6 CONCLUSION

As we continue to develop antiepileptogenic and disease‐modifying epilepsy therapies that move closer and closer to clinical testing, it will be important to simultaneously develop optimal biomarkers and trial methodologies that will be scientifically rigorous and accurate, and also acceptable for regulatory approvals. There is no doubt that in the next decade we will see trials, and possibly even approved products. This will be a major benefit to prevent or ameliorate a devastating disease.

## CONFLICT OF INTEREST

J. French receives salary support from the Epilepsy Foundation and for consulting work and/or attending Scientific Advisory Boards on behalf of the Epilepsy Study Consortium for Adamas, Aeonian/Aeovian, Alterity Therapeutics Limited, Anavex, Arkin Holdings, Arvelle Therapeutics, Inc, Athenen Therapeutics/Carnot Pharma, Baergic Bio, Biogen, BioXcel Therapeutics, Cavion, Cerebral Therapeutics, Cerevel, Corlieve Therapeutics, Crossject, CuroNZ, Eisai, Eliem Therapeutics, Encoded Therapeutics, Engage Therapeutics, Engrail, Epalex, Epihunter, Epiminder, Equilibre BioPharmaceuticals, Fortress Biotech, Greenwich Biosciences, GW Pharma, Janssen Pharmaceutica, Knopp Biosciences, LivaNova, Longboard Pharmaceuticals, Lundbeck, Marinus, Mend Neuroscience, Merck, NeuCyte, Inc, Neumirna Therapeutics, Neurocrine, Neuropace, Otsuka Pharmaceutical Development, Ovid Therapeutics Inc, Passage Bio, Praxis, PureTech LTY Inc, Redpin, Sage, SK Life Sciences, Sofinnova, Stoke, Supernus, Synergia Medical, Takeda, UCB Inc, West Therapeutic Development, Xenon, Xeris, Zogenix, Zynerba. J. French has also received research support from the Epilepsy Study Consortium (Funded by Andrews Foundation, Eisai, Engage, Lundbeck, Pfizer, SK Life Science, Sunovion, UCB, Vogelstein Foundation) Epilepsy Study Consortium/Epilepsy Foundation (Funded by UCB), GW/FACES, and NINDS. She is on the editorial board of Lancet Neurology and Neurology Today. She is Chief Medical/Innovation Officer for the Epilepsy Foundation. She has received travel reimbursement related to research, advisory meetings, or presentation of results at scientific meetings from the Epilepsy Study Consortium, the Epilepsy Foundation, Arvelle Therapeutics, Inc, Biogen, Cerevel, Engage, Lundbeck, NeuCyte, Inc, Otsuka, Sage, UCB, Xenon, Zogenix. Sergiusz Jozwiak has received consulting, speaker, or advisory board fees from Novartis, Biogen, Eisai, and GW Pharma. Martina Bebin receives NINDS grant support and is a subinvestigator for Greenwich Biosciences. Martina Bebin is on advisory committees for Neurelis, Greenwich Biosciences, Aquestive Therapeutics, REGENXBIO. Adam Hartman is an Editorial Board member for Neurology and a consultant for Best Doctors/Teladoc, Inc Pavel Klein has received consulting, speaker, or advisory board fees from Arvelle, Aquestive, Biogen, Eisai, Engage Therapeutics, Greenwich Pharmaceuticals, Lundbeck, Neurelis, SK Life Science, Sunovion, and UCB Pharma. He receives grant support from DOD/CURE. He is a member of medical advisory board of Stratus‐Alliance, of Scientific Advisory Board of OB Pharma, and the CEO of PrevEp. Arvelle (cenobamate), Eisai (perampanel, rufinamide), GW Pharma (cannabidiol), Lundbeck (clobazam), Neurelis (Valtoco), Novartis (everolimus), SK Life Science (cenobamate), Sunovion (eslicarbazepine), and UCB Pharma (lacosamide, brivaracetam) manufacture, or sell drugs evaluated or mentioned in this study. Paul Vespa receives NINDS funding and is a consultant for Ceribell and UCB Pharmaceuticals. The remaining authors have no conflicts of interest to disclose. We confirm that we have read the Journal's position on issues involved in ethical publication and affirm that this report is consistent with those guidelines.

## DISCLAIMER

This report does not represent the official view of the National Institute of Neurological Disorders and Stroke (NINDS), the National Institutes of Health (NIH), or any part of the US Federal Government. No official support or endorsement of this article by the NINDS or NIH is intended or should be inferred.
